# The effect of military clothing on gunshot wounding patterns in gelatine

**DOI:** 10.1007/s00414-018-1972-8

**Published:** 2018-11-28

**Authors:** Tom Stevenson, Debra J. Carr, Sarah A. Stapley

**Affiliations:** 1 Impact and Armour Group, Centre for Defence Engineering, Defence Academy of the United Kingdom, Cranfield University, Shrivenham, SN6 8LA UK; 2 Cranfield Forensic Institute, Defence Academy of the United Kingdom, Cranfield University, Shrivenham, SN6 8LA UK; 3 Present Address: Defence and Security Accelerator, Porton Down, Salisbury, SP4 0JQ UK; 4 Royal Centre for Defence Medicine, ICT Building, Research Park, St Vincent Drive, Birmingham, B15 2SQ UK

**Keywords:** Gunshot, Wounding, Clothing, Gelatine, Military, AK47, AK74

## Abstract

With no two gunshot wounds (GSW) being the same, novel research into wound ballistics is challenging. It is evident that the majority of previous wound ballistic research has been conducted without the presence of clothing. Whilst the effect of clothing on wound contamination has been explored, there is a paucity of literature examining the effect of clothing on GSW patterns. The aim of this study was to test the effect of Multi-Terrain Pattern (MTP) UK military clothing on GSW patterns within calibrated blocks of 10% by mass gelatine, using two types of ammunition commonly used in recent conflicts—7.62 × 39 mm and 5.45 × 39 mm. In total, 36 blocks were shot, 18 by each projectile type, further divided into 6 with no clothing layers (C_nil_), 6 with a single clothing layer (C_min_) and 6 with maximum clothing layers (C_max_) worn on active duty. Blocks were analysed with high-speed video and dissection to capture measurements of damage, and results compared using analysis of variance (ANOVA). Results showed significantly different damage measurements within blocks with C_max_ for both ammunition types compared to the other clothing states. This may result in GSWs that require more extensive surgical management, inviting further study.

**Electronic supplementary material:**

The online version of this article (10.1007/s00414-018-1972-8) contains supplementary material, which is available to authorized users.

## Introduction

During the recent Iraq and Afghanistan wars (2003–2014), the UK military suffered 723 gunshot wound (GSW) casualties with 177 fatalities and 546 survivors leading to a substantial clinical burden [1]. Historical review has demonstrated that clinical lessons learned from previous conflicts are often lost, leading to potentially avoidable higher morbidity amongst casualties [2, 3]. It is therefore paramount that studies are undertaken using appropriate methods to continually test existing theory and research conducted over the last century, and help develop novel strategies to further understand wound ballistics. This may improve patient outcomes [4], and ultimately retain corporate knowledge gained previously and pass it on to the next generation of clinicians.

The majority of existing GSW research has been conducted on naked animals or cadavers or bare tissue simulants [e.g. 5–16]. Whilst the effects of clothing on GSW have been examined with respect to contamination [e.g. 17–21], there remains a paucity of literature examining the effect of clothing on the wounding patterns; exceptions include separate works by Kieser, Carr, Mabbott and Mahoney [22–25].

Gelatine has been used for wound ballistic research since the early twentieth century, with different concentrations and configurations depending on the aims of the respective studies [26–34]. Research conducted at the Letterman Institute in the USA re-validated the use of gelatine as comparable to live swine thigh muscle tissue with regard to its response to ballistic testing. This can offer a useful way to visualise GSW profiles from different ammunition types [35–37]. Studies from the last 5 years have examined the difference in gelatine concentrations to determine positive and negative attributes for certain uses within wound ballistic research [24, 38, 39]. The use of gelatine in wound ballistic research has also recently been summarised and highlights the difficulty in accurately reproducing wounding patterns despite controlling as many variables as possible [4]. With clinicians often stating that no two GSWs are ever the same [40], such modelling poses a real challenge to the researcher in order to achieve their aim. As well as gelatine, other media used in ballistic modelling include ballistic soap, cadaveric animal and human tissue, live animal tissue and other synthetic tissue simulants, all of which have been subject of recent review [41].

It helps to consider wounding patterns that occur within gelatine blocks in several different stages which are explored in greater detail within Kneubehl’s comprehensive text “Wound Ballistics” [42] and are summarised as follows:

- *Temporary cavity*: The temporary cavity is formed following transfer of kinetic energy (KE) from the projectile to the gelatine. The KE causes the gelatine to radially accelerate away from the projectile, generating negative pressure, drawing air in from the entrance (and/or exit) wound and forming the temporary cavity. The size of the temporary cavity can vary along the wound track and is determined by the amount of KE being transferred, which is in turn determined by the contact surface area of the projectile. Should the projectile yaw, expand and/or fragment, its contact surface area with the target is increased at that point, causing an increase in drag coefficient resulting in more rapid deceleration, and leads to greater delivery of KE and thus greater temporary cavitation. The temporary cavity, by the physical properties associated with its formation, is multiple times larger than the permanent cavity left behind.
- *Permanent cavity:* This consists of the track formed by the projectile crushing and cutting its way through the gelatine, and the damage caused by the formation and collapse of the temporary cavity. When a projectile of a certain type (for example military projectiles, such as 7.62 × 39 mm) strikes a target nose on, an initial narrow wound channel (i.e. the neck length) is created whilst the projectile is still travelling symmetrically (and is arguably of the greatest surgical relevance as marginal to no surgical debridement of tissues is required [14, 43]). There is little damage seen as the projectile’s contact surface area with the gelatine is at its minimum. With a longer neck length, the projectile may go on to exit the target before yawing, and as such takes the majority of KE with it, leaving a potentially smaller and simpler wound profile behind—again, clinically, this is important and will be revisited within the discussion section of this paper. It should be noted that other ammunition types, such as expanding projectiles, may have little to no neck length at all with extensive cavitation seen. Other projectile types, such as ball bearings, are of a uniform spherical shape so will not yaw and also do not deform in shape and may only leave a narrow track following minimal temporary cavitation. Knowledge of these properties helps identify wound patterns attributable to those projectile types.

Understanding the wounding pattern helps facilitate calculation of the area or volume of gelatine damage seen. With respect to what measurements are relevant, this is variable and is determined by the aim of the study. Examples include measuring the depth of penetration (DoP) of projectiles into the gelatine block, the dimensions of the temporary cavity using high-speed video (HSV), the dimensions of the permanent cavity, the distance from entry to which the projectile yaws 90° and imaging of wound tracks using medical imaging modalities [4, 22, 24, 25, 36, 44–48].

The types of ammunition used in ballistic modelling are dependent on what the subject for study demands. Typically for modelling directed at the use of military grade firearms, high-velocity rifle ammunition is used, e.g. 7.62 × 39 mm, 7.62 NATO (7.62 × 51 mm), 5.45 × 39 mm and 5.56 NATO (5.56 × 45 mm). This list is by no means exhaustive; there are numerous studies examining different projectile types, such as steel ball bearings [24, 49]. With physical, mechanical and ballistic properties of ammunition varying widely but rarely being discussed within the literature, it is preferential to use a single quarantined batch of required ammunition types and, if necessary, identify composition and microhardness [4].

The ballistic protective performance of winter issue military clothing has been reported; however, this examined the failure of the clothing rather than any wounding patterns seen as a result of ballistic impact [50]. A study of rifle ammunition effects on tissues considered anaesthetized pigs clothed in Finnish military uniforms however made no comment on the effect of the presence of the clothing on the wounding patterns [51].

More recently published was a study that showed the presence of a layer of denim on a model of a deer femur embedded in 20% (by mass) gelatine led to an increase in the risk of indirect femoral fracture when shot by 5.56 NATO ammunition [22], followed by an increasing interest in examining clothing effects on wounding in ballistic research [e.g. 4, 20, 21, 23, 44, 45]. Published research has demonstrated that intermediate layers (clothing or other personal protective equipment) can affect damage sustained by a gelatine block during ballistic testing [e.g. 22, 23, 25, 44].

Whilst it can be acknowledged that previous research on naked tissue and tissue simulants has been conducted, it is evident that professional troops going into active conflict in the modern era will be appropriately clothed. With respect to UK service personnel, that clothing is typically in the form of standard issue Multi-Terrain Pattern (MTP) clothing, with different layers worn depending on the climate and the nature of the operations being conducted. The effect of military clothing on wounding patterns does not appear to have previously been examined.

The aim of the current study was to characterise the effect of military clothing on GSW patterns in blocks of 10% by mass-calibrated gelatine using 7.62 × 39 mm and 5.45 × 39 mm ammunition, whilst considering the clinical relevance of the results.

## Materials and methods

Ethical approval for this work was granted via Cranfield University Research Ethics System (CURES/3579/2017).

### Materials

Thirty-six blocks of 10% (by mass) gelatine were made in batches of six from type 3 photographic grade gelatine (GELITA® AG, Uferstraβe 7, 69412, Eberbach, Germany; bloom strength 263). Moulding tins had inside dimensions of 250 × 250 × 500 mm, with a 1° taper to facilitate set gelatine removal [44]. The blocks were conditioned at 4 °C for 24 h after setting.

**Fig. 1:**
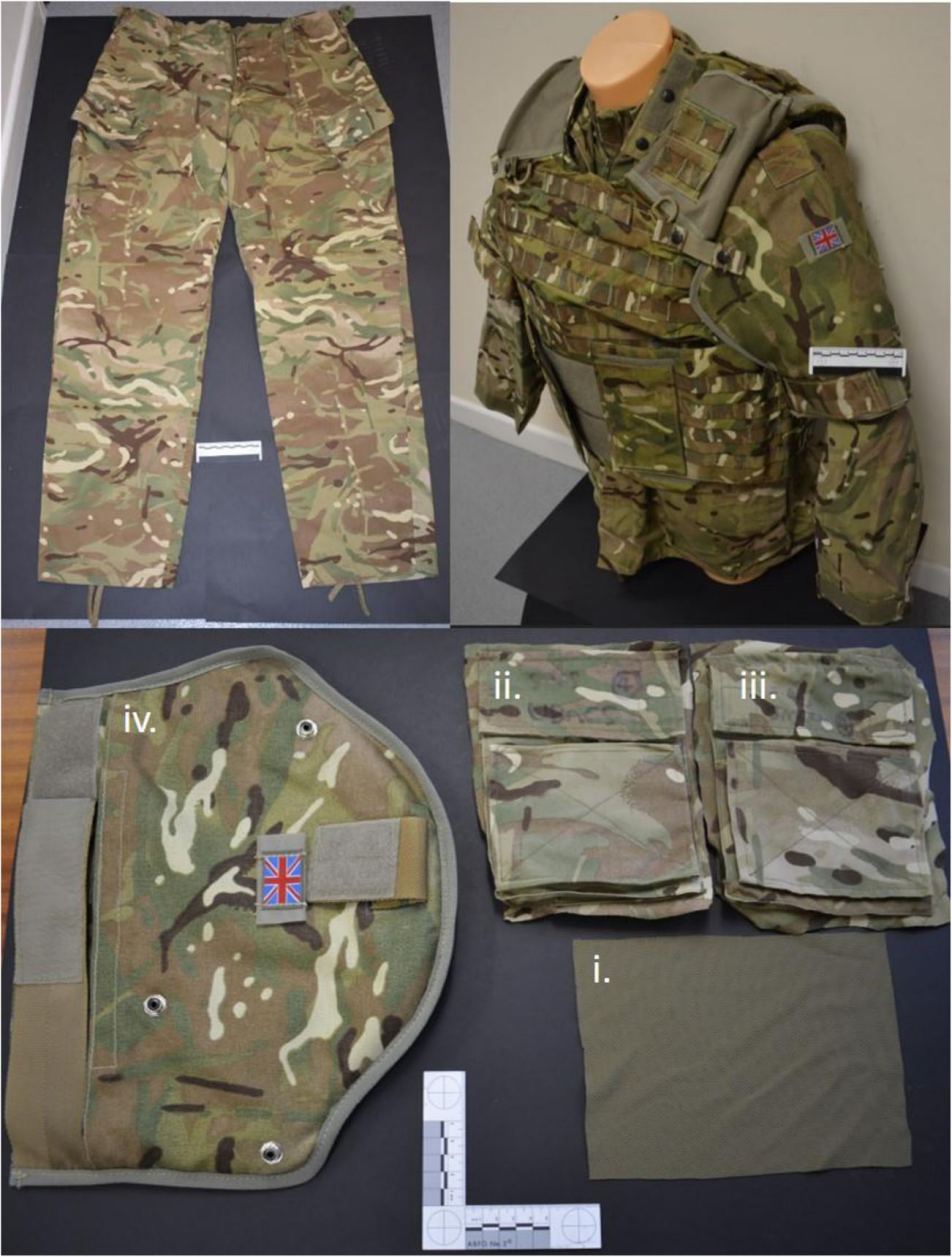
Examples of MTP clothing used. *clockwise from top left* MTP trousers. *top right* T-shirt, UBACS, smock and brassard as worn by service personnel. *bottom* (i) T-shirt, (ii) UBACS, (iii) smock and (iv) brassard layers prepared for testing

The MTP clothing selected for investigation was divided into different states to represent the minimal and maximal layers worn globally by UK personnel on combat and front-line duties. Firstly, bare blocks of gelatine or a zero clothing state (C_nil_) was used for a control. The minimal clothing state (C_min_) was represented by a single clothing layer taken from MTP trousers^1^ (*n* = 6) (Fig. 1). Finally, the maximal clothing state (C_max_) involved several layers of clothing including a base layer standard issue t-shirt^2^ (*n* = 6), upper arm sleeve pocket of Under Body Armour Combat Shirt (UBACS)^3^ (*n* = 6), the upper arm sleeve pocket of an MTP smock jacket^4^ (*n* = 6) and finally a brassard (upper arm protection). The brassard consisted of a fragment protective filler^5^ manufactured from a para-aramid fabric, sealed in a light- and water-resistant cover. This was inserted into an outer carrier^6^ which attaches to the body armour torso as part of the OSPREY body armour system (*n* = 12 for both items) (Fig. 1) [52]. All clothing, excluding the brassards, was laundered (following procedure 8A of British Standard EN ISO 6330: 2001) by washing six times before drying informed by the care label provided in the garment and to ensure the removal of any finishing treatments and dimensional stability of the fabric [53].^7^

Fabric samples of individual clothing layers were analysed (*n* = 5) in order to characterise their physical properties. Mass per unit area and thickness of the samples were measured [54, 55], using Oxford A2204 scales to measure mass and a Mitutoyo C1012MB thickness gauge to measure thickness of the MTP trouser single layer for C_min_, and the individual layers of the t-shirt, UBACS and smock as part of C_max_. The brassard and all combined layers for C_max_ were measured using Mettler PE16 scales for mass and a Shirley Thickness Gauge (Shirley Developments Ltd., 87,137) for thickness.

**Fig. 2:**
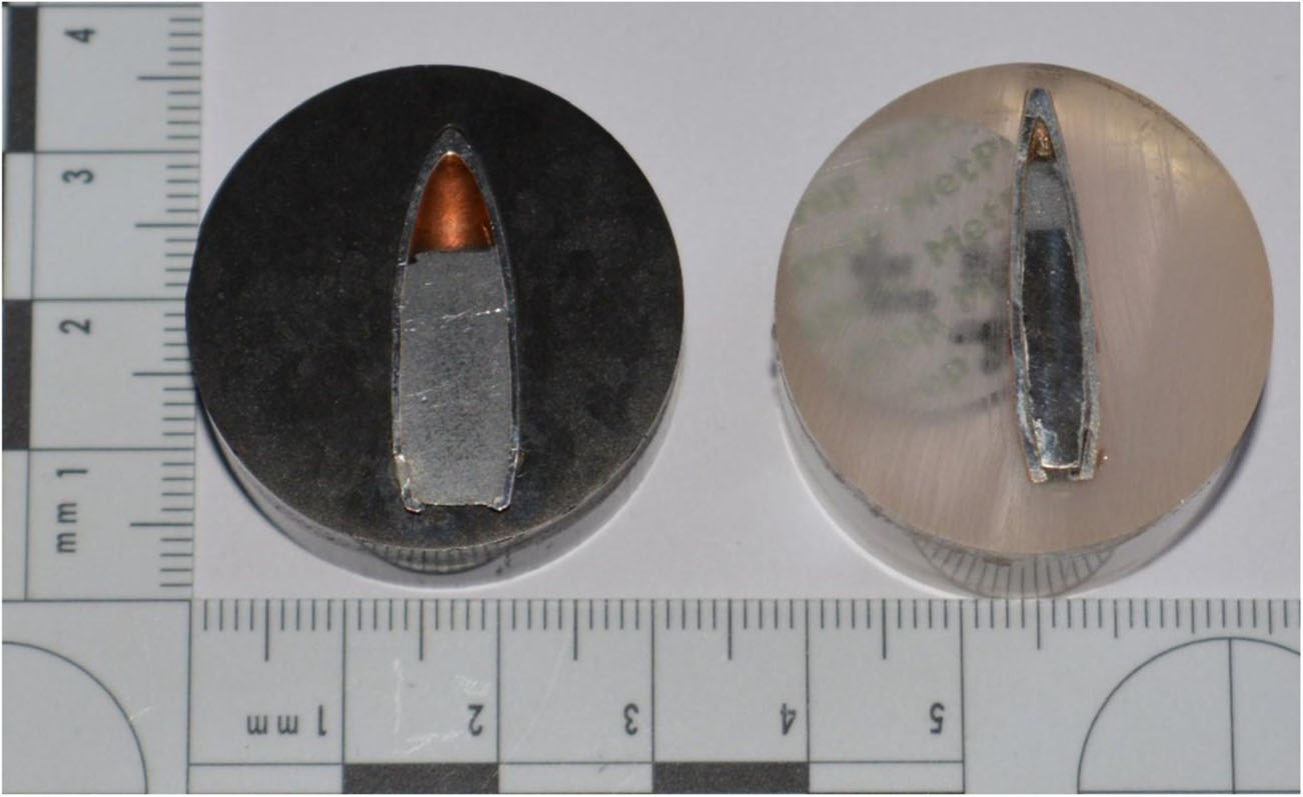
Mounted sections of 7.62 mm (*left*) and 5.45 mm (*right*) projectiles

In recent conflicts that UK Armed Personnel have participated in, a wide range of weapons systems were used. Two common weapons systems available in Iraq and Afghanistan (2003–2014) that were used against UK Armed Forces were the AK47 and the AK74 [56, 57]. The ammunition used with these weapons systems is 7.62 × 39 mm and 5.45 × 39 mm, respectively. Therefore, these two types of ammunition were used in the current study. To help control the variability in ammunition batch production, batches of ammunition were quarantined for this study: 7.62 × 39 mm (7.62 × 39 mm Wolf Hunting Cartridges; lead core, 122 grain full metal jacket, lot number F-570, made in Russia, 2006) and 5.45 × 39 mm (5.45 × 39 mm; mild steel core, 53 grain full metal jacket, lot number 539–04, made in Russia, 2004) (Fig. 2). Hardness was determined by sectioning and encapsulating projectiles in epoxy resin (*n* = 3), using a Struers Rotopol 15 to polish the sample projectiles, and an Indentec Highwood microscope with diamond tipped load point to measure hardness. Elemental composition was determined using a Hitachi SU3500 scanning electron microscope with EDAX analysis and TEAM software.

### Methods

**Fig. 3:**
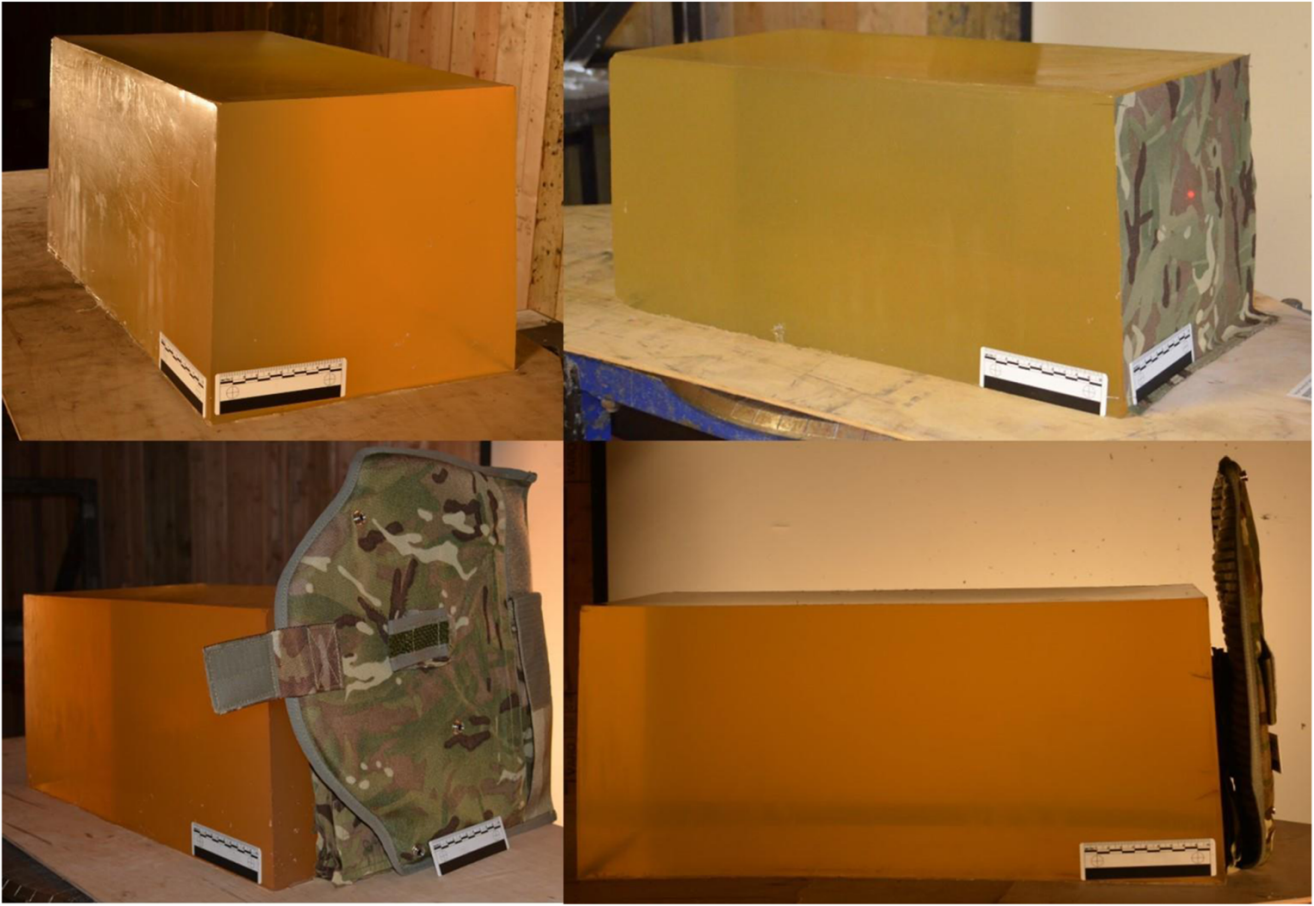
*clockwise from top left* C_nil_ oblique view, C_min_ oblique view, C_max_ side view, C_max_ oblique view

Fabric samples for C_min_ were cut from laundered MTP trousers (250 × 250 mm) and pinned to the front face of the gelatine blocks (Fig. 3). Fabric samples for C_max_ were measured and cut in relation to the upper sleeve pocket size on the UBACS and smocks (200 × 150 mm), and placed in layers with the t-shirt layer innermost, then UBACS, smock and finally with the brassard then placed over the top of the other layers (Fig. 3).

An indoor small arms range was used to fire projectiles from a number 3 proof housing, where the end of the barrel was situated at 10 m from the target. The gelatine was calibrated by firing a 5.5 mm ball bearing into each block; DoP was measured and compared to previously published studies to ensure validity of the blocks used in this series of experiments [25, 38, 58]. Each block was then shot once with the test projectiles. Eighteen blocks were shot with 7.62 mm projectiles and the remaining 18 blocks were shot with 5.45 mm projectiles. Six blocks for each ammunition type had either C_nil_, C_min_ or C_max_ added to the impact face.

**Fig. 4:**
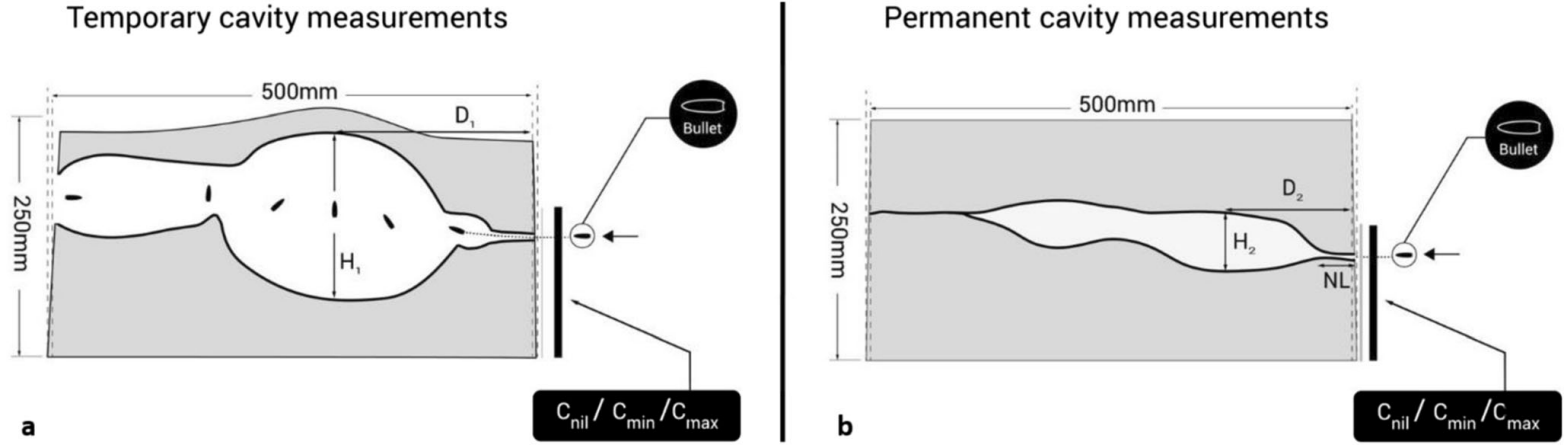
**a** Temporary cavity measurement schematic. **b** Permanent cavity measurement schematic

The impact velocity for each projectile was measured using Doppler radar (Weibel W700). HSV using a Phantom V1212 video camera (frames per second = 37,000, shutter speed = 5 μs, resolution = 512 × 384) allowed visualisation of the wounding pattern and to record the formation of the temporary cavity. Measureable parameters were taken from the HSV of this phenomenon using Phantom Software (Visions Research, Phantom Camera Control Application 2.6). These parameters included maximum height of the temporary cavity (H1) and distance to the maximum height of the temporary cavity (D1), where the latter corresponded to the point where the projectile was at maximum yaw of 90° [36] (e.g. Fig. 4a). Temperature of the gelatine blocks was recorded after shooting using a calibrated digital thermometer. Black food colouring was poured in via entrance wounds of the gelatine blocks to visually highlight wounds. Gelatine blocks were then dissected and any fragmentation of the projectiles noted and recovered. The damage to the gelatine block was photographed using a Canon D5100 Digital SLR camera (S/N 6773411). The parameters of damage measured were maximum height of the permanent cavity (H2), distance to maximum height of the permanent cavity (D2) and neck length (NL) (e.g. Fig. 4b).

The International Business Machine Corporation’s Statistical Package for Social Services version 24 (IBM SPSS Statistics v24) analysis of variance (ANOVA) was used to determine the effect of the different clothing states^8^ on H1, D1, H2, D2 and NL. The two ammunition types were considered together, and homogeneity of variance and normality of data were confirmed with a significance level of 0.05 applied. Significant differences due to ammunition type and/or clothing condition were identified using Tukey’s honest significant difference (HSD) test. Main effects and significant interactions only are discussed in the “Results” section.

## Results

**Fig. 5:**
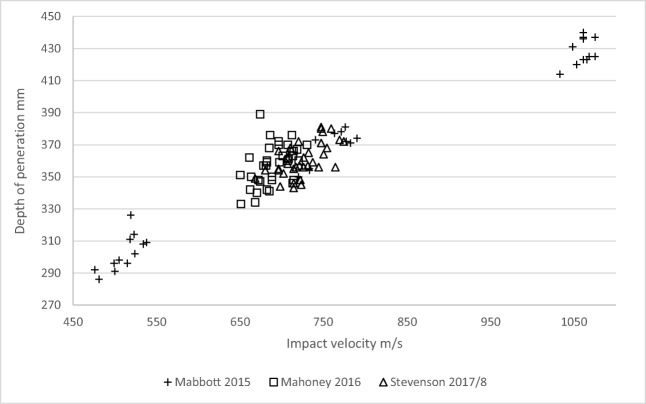
10% gelatine (4 °C) calibration data (Stevenson 2018 current study, compared to historical data [44, 59])

Calibration of the gelatine blocks using 5.5-mm-diameter ball bearings (mean impact velocity of 725 m/s, SD = 26 m/s; mean DoP = 361 mm, SD = 11 mm) was similar to previously collected data giving confidence in the consistency of the blocks (Fig. 5). Mean impact velocity for the 7.62-mm projectiles was 648 m/s (SD = 8 m/s) and for the 5.45-mm projectiles was 883 m/s (SD = 14 m/s). Mean temperature of the gelatine blocks after testing was 6.8 °C (SD = 1.6 °C).

Ammunition characteristics are given in Table 1. As expected, both projectiles were jacketed in steel with copper washes and the lead core of the 7.62 mm projectile was softer than the steel core of the 5.45 mm projectile which had a soft lead tip.

**Table 1:** Characteristics for 7.62 × 39 mm and 5.45 × 39 mm ammunition

Projectile type		Core hardness (Hv)	Jacket hardness (Hv)	Tip hardness (Hv)
7.62 mm	Mean	7.39 (*n* = 3)	184.57 (*n* = 5)	N/A
	SD	0.86	9.91	N/A
	Composition	Lead, antimony	Steel (with internal/external copper wash)	N/A
5.45 mm	Mean	820.90 (*n* = 3)	188.90 (*n* = 5)	4.58 (*n* = 2)
	SD	15.85	15.41	1.05
	Composition	Steel	Steel (with internal/external copper wash)	Lead

Mass per unit area and thickness for C_min_ and C_max_ are given in Table 2. The single trouser layer used for C_min_ was thinner and lighter than the combined layers used for C_max_ as would be expected. The C_max_ thickness and mass per unit area was calculated using all layers together, as would be worn in reality.

**Table 2:** Mass per unit area and thickness for clothing states

Clothing state		Mass per unit area (g/m^2^)	Thickness (mm)
C_min_	Mean	191.14	0.43
	SD	1.76	0.02
C_max_	Mean	7735.17	32.26
	SD	86.02	0.97

Seventeen of the 7.62 mm projectiles and 10 of the 5.45 mm projectiles exited the blocks across all clothing conditions. For the 7.62 mm projectiles, all exits were via the rear face. For the 5.45 mm projectiles, one of the projectiles exiting exited via the rear face, four via the right face (as viewed from the impact face) and five exited via the top face. For projectiles that were retained, the DoP was measured: for the one 7.62 mm projectile retained, the DoP was 484 mm; for the eight 5.45 mm projectiles retained, the mean DoP was 423 mm (SD = 14 mm), though it was noted from the HSV that all those retained 5.45 mm projectiles except for one would have exited via the bottom face but instead were retained due to ricochet off the table the block was mounted on. The retained 7.62 mm projectile was in a gelatine block with C_nil_, and the one truly retained 5.45 mm projectile (which did not ricochet of the base table) was in a block with C_max_; therefore, the clothing state was unlikely to have influenced the rate of projectile retention.

**Fig. 6:**
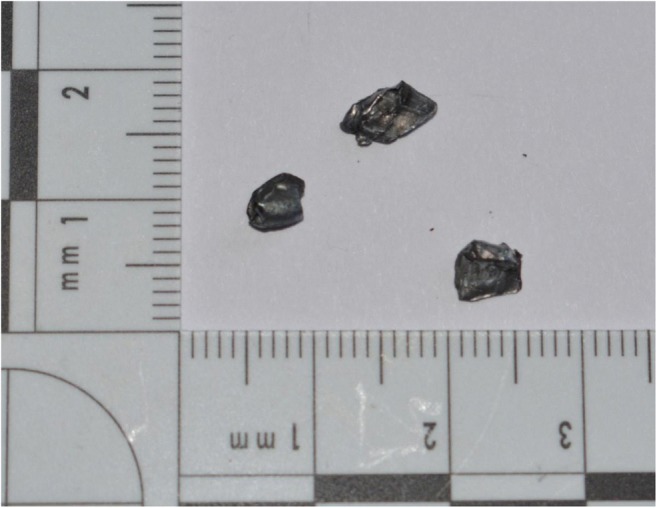
Typical fragmentation recovered from gelatine shot by a 7.62 mm projectile

Seventeen of the 7.62 mm projectiles fragmented; 94% of those fragments were retained within the blocks and four of the 17 shots that fragmented had more than one fragment, with a maximum of three fragments seen (Fig. 6). Mass of fragments varied from 0.04 to 0.61 g (mean = 0.30 g, SD = 0.16 g). The difference seen in the number of projectiles that fragmented or the number of fragments seen amongst blocks with or without clothing layers was either non-existent or too small for statistical comparison. The mean DoP of the fragments was 350 mm (SD = 97 mm). None of the 5.45 mm projectiles fragmented. This data suggests that the clothing state did not influence the fragmentation of the projectiles, and that this was more likely due to the composition and construction of each ammunition type and the forces applied to the projectile during the interaction with the target.

The dimensions collected for the damage caused by the temporary and permanent cavities to the gelatine blocks are summarised in Table 3.

**Table 3:** Mean, standard deviation (SD) and coefficient of variation (CV) for dimensions measured

	NL			D1			H1			D2			H2		
Projectile / clothing state	Mean (mm)	SD (mm)	CV (%)	Mean (mm)	SD (mm)	CV (%)	Mean (mm)	SD (mm)	CV (%)	Mean (mm)	SD (mm)	CV (%)	Mean (mm)	SD (mm)	CV (%)
7.62 mm/C_nil_	72.5	41.6	57.3	195.3	31.0	15.9	184.7	21.7	11.7	199.7	54.5	27.3	132.7	30.5	23.0
7.62 mm/C_min_	74.5	58.3	78.3	191.0	63.4	33.2	192.8	13.6	7.0	178.0	69.8	39.2	133.2	29.0	21.8
7.62 mm/C_max_	26.3	22.0	83.6	153.0	30.3	19.8	204.0	28.4	13.9	135.0	38.0	28.0	122.0	17.3	14.2
5.45 mm/C_nil_	71.7	43.8	61.1	179.0	39.9	22.3	211.3	29.8	14.1	152.7	47.0	30.8	134.7	5.0	3.7
5.45 mm/C_min_	51.0	12.1	23.8	182.0	18.5	10.2	181.7	8.5	4.7	163.0	44.7	27.4	126.7	7.6	6.0
5.45 mm/C_max_	9.7	8.2	84.5	116.0	10.0	8.7	173.0	7.5	4.3	108.0	22.1	20.5	128.0	9.4	7.3

When considering the effect of clothing state on data variability from Table 3 for each ammunition type, no clear trends were observed except for the following:

- 7.62 mm—increasing variability in NL with increasing clothing state and decreasing variability in H2 with increasing clothing state
- 5.45 mm—increasing variability in H2 with increasing clothing state and decreasing variability in D1, H1 and D2 with increasing clothing state.

ANOVA results are given in Table 4 below; data subgroups identified by Tukey’s HSD are also included.

**Table 4:** ANOVA results

Measurement	ANOVA effects (*F*-statistic, *p* value)		Data subsets found (Tukey’s HSD)	
	Clothing state	Ammunition type	Group 1	Group 2
NL	*F*_2, 30_ = 7.39, *p* ≤ 0.01	*F*_1, 30_ = 3.10, *p* = NS	C_max_	C_min_, C_nil_
D1	*F*_2, 30_ = 7.12, *p* ≤ 0.01	*F*_1, 30_ = 6.05, *p* ≤ 0.05	C_max_	C_min_, C_nil_
H1	*F*_2, 30_ = 4.88, *p* ≤ 0.05	*F*_1, 30_ = 6.96, *p* ≤ 0.05	C_max_, C_min_	C_min_, C_nil_
D2	*F*_2, 30_ = 4.26, *p* ≤ 0.05	*F*_1, 30_ = 6.75, *p* ≤ 0.05	C_max_, C_min_	C_min_, C_nil_
H2	*F*_2, 30_ = 0.74, *p* = NS	*F*_1, 30_ = 0.26, *p* = NS	No subgroups identified	

In all measurements apart from H2 it was demonstrated that the clothing state of C_max_ led to significantly different measurements when compared to C_nil_. In the cases of NL and D1 measurements, C_max_ also led to significantly different measurements when compared to C_min_.

## Discussion

**Fig. 7:**
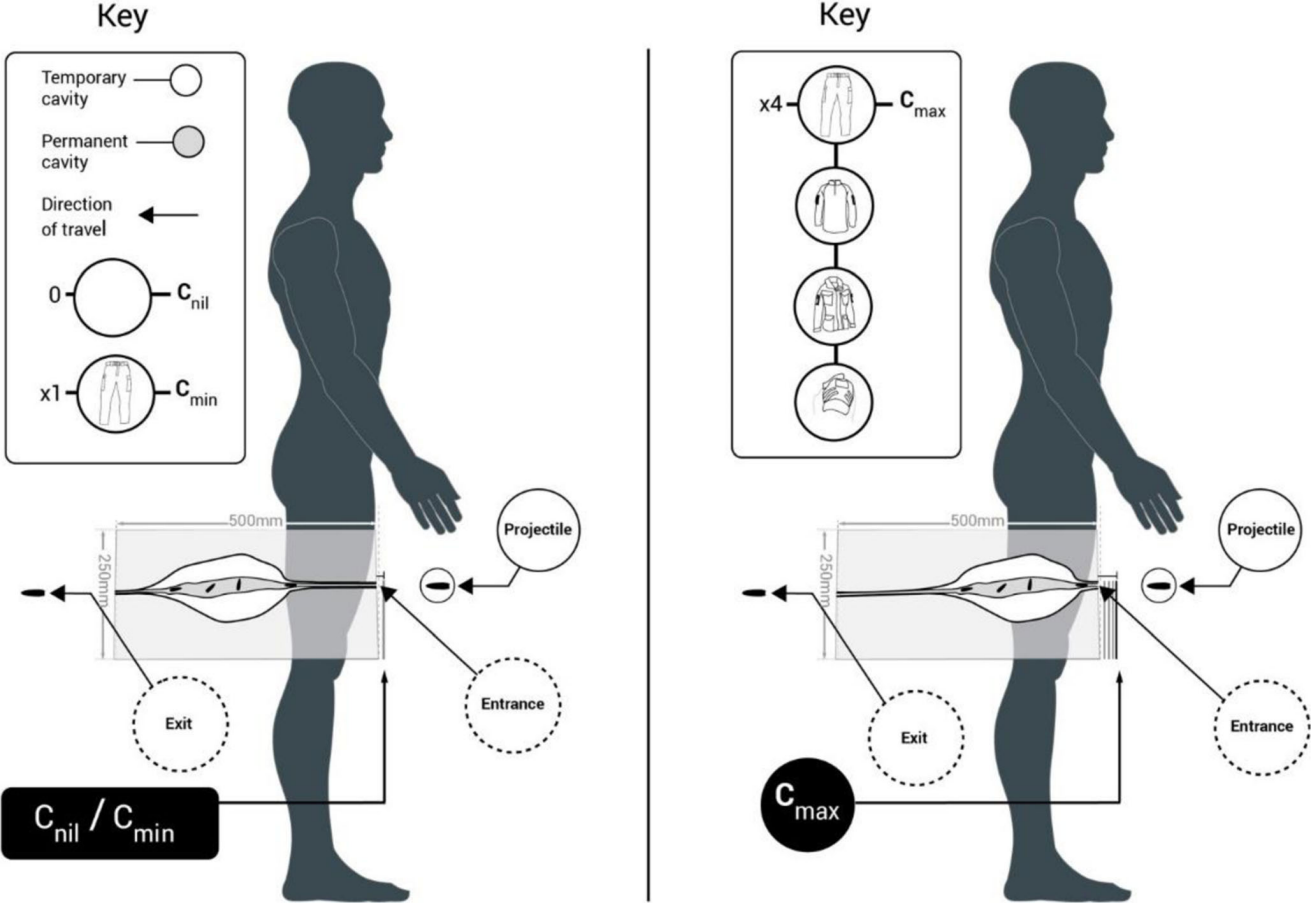
Anatomical overlay of GSW patterns—C_nil_ and C_min_ (*left*) and C_max_ (*right*)

The clinical effects of a GSW will be dictated by both the ammunition effects and clothing effects together. When compared to an anatomical overlay (Fig. 7), a projectile which might have otherwise passed through a limb before yawing significantly would yaw sooner within that limb due to C_max_. This would cause temporary cavitation to occur earlier and impart a greater amount of KE and subject those tissues to greater deformative stress. Crucially, the resultant effect would undoubtedly require an increased level of surgical intervention, bringing with it the associated risks of carrying out such surgery to the patient.

Interestingly, the effect of the ammunition on the temporary cavity varied with clothing state. That the temporary cavity height was smaller where 5.45 mm projectiles are used with C_max_ does not matter, because the damage still occurred earlier within the wound tract and was still greater than that seen within the neck length which exists at the same position in blocks with C_min_ and C_nil_ (Fig. 7 and Table 3).

Introducing a layer of any material, such as clothing, between a projectile and its target brings further potential to alter the symmetry of flight of that projectile. The effect of intermediate layers has been reported previously, though not specifically on the effect of military clothing [22, 23, 25, 44]. The presence of military clothing layers could mean an increased chance of the projectile yawing away from its central axis by several degrees within the microseconds following interaction with the material but before striking its target. This would increase the contact surface area of the projectile striking the target and thus lead to higher KE transfer and potentially subject that tissue to greater damage earlier on in the projectile/target interaction. This holds particular relevance with respect to the NL measurements, where the NL region of a body limb wound typically requires less surgical intervention. This translates to the NL being a key measurement of damage; the longer it is, the more likely the projectile has exited before imparting much of its KE and the chance is greater for a wound pattern requiring less clinical intervention.

The fragmentation of projectiles seen was exclusive to 7.62 mm, and most likely occurred due to the composition and construction of those projectiles rather than due to the clothing state. This was supported by the fact that the only 7.62 mm projectile not to fragment had passed through C_max_, and by the fact that none of the 5.45 mm projectiles fragmented within blocks of all three clothing states. As the fragments were extremely small, the overall damage they contributed within the wounding patterns was negligible. Clinically, removing such fragments has the potential to cause more harm than benefit so, unless causing direct neurovascular injury, operating clinicians sometimes opt to leave them in situ.

Of qualitative interest was that the visual inspection of the HSV data showed a wounding pattern seen in real time that was grossly peculiar to each ammunition type irrespective of the presence of clothing layers as shown in the animations (Online resources 1 and 2), though this observation in itself was not further quantified or statistically tested beyond the above results.

Microhardness and elemental analysis results suggested that both types of ammunition were manufactured consistently. This was also true of the fabric analysis results with regard to the use of the different layers of MTP for the relevant clothing states. To the knowledge of the authors of this work, the effect of UK military clothing on GSW patterns has not previously been considered within existing literature.

### Limitations

One of the main limitations of this model is that gelatine is a synthetic medium and as such cannot in any way allow comment on tissue viability within such wounds as re-created in this study. As such, a number of assumptions have to be made when considering the clinical relevance of wounding patterns within synthetic modelling. It stands to reason that where maximal temporary cavitation occurs, tissues in a live subject would be exposed to greater stress and potential damage compared to an area in the tissue where temporary cavitation is minimal, i.e., the neck length, though without live tissue testing under the same conditions, it cannot be proven beyond the anecdotal experience of authors whom have seen such injuries within their clinical practice and can provide comment.

Another limitation is clothing type. Though in regular use on day-to-day active service for the UK military, the MTP clothing selected for this testing does not appear to have been previously discussed. This means that there is no way to compare the results of this study directly with other studies at this time, although it does offer a point of comparison for future studies.

The ammunition types chosen also are a limitation where troops can be exposed to a plethora of different ammunition types during conflicts, depending entirely on the enemy logistical infrastructure. Even ammunition of the same type may have different physical properties and characteristics due to being of different batches or manufactured in different countries [4].

Other limitations include the fixed engagement distance and controlled projectile velocities; it is unlikely to expect that GSWs are sustained regularly at muzzle velocity with a projectile flying symmetrically in all combat scenarios. Engagement distances with the enemy will always vary, as will the subsequent velocity and potential asymmetry of the projectile in flight upon striking the target; thus, the behaviour of the ammunition being fired is determined due to the number of external influences prior to impact. This further reinforces a need to control variables as a measure of scientific rigour to allow accurate testing, hence to why the above testing conditions were set, to try and minimise the amount of variability beyond that which was to be examined.

## Conclusion

C_max_ significantly affected the damage sustained by a gelatine block shot by 7.62 mm or 5.45 mm projectiles raising the possibility of a more complicated surgical intervention being required for human casualties wearing such clothing combinations. C_min_ did not affect the damage sustained by a gelatine block shot by 7.62 mm or 5.45 mm projectiles. Neither iteration of MTP clothing layers appeared to affect the propensity of bullet fragmentation, retention nor the path which was taken by the projectile after entering the gelatine block, though the latter was extremely difficult to quantify from the data collected.

## Electronic supplementary material

Online resource 1 – Typical GSW profile in bare gelatine block from 7.62 mm projectile (MP4 1999 kb)

Online resource 2 – Typical GSW profile in bare gelatine block from 5.45 mm projectile (MP4 2385 kb)

## Footnotes

1 Trouser, combat, warm weather MTP—NATO stock number (NSN): 8415–99–317-8313

2 T-shirt, combat, anti-static, light olive—NSN: 8415–99–813-3258

3 Shirt, UBACS, MTP—NSN: 8415–99–317-8402

4 Smock, combat, windproof, MTP—NSN: 8415–99–317-8386

5 Filler Osprey Mk 2—NSN: 8470–99–480-8055

6 Osprey MKIVA (MTP) cover brassard—NSN: 8470–99–684-4613-4

7 BEKO washing machine (model number WM84125W) used on a cotton cycle lasting 79 min per cycle with a water temperature of 40 °C; BEKO tumble dryer (model number DSV64W) used on a 60-min cycle at the standard factory set temperature (not listed).

8 The effects of *C*_min_ on GSW patterns were presented as a poster at the 30th International Symposium on Ballistics [45].
